# Low Detection Limit and High Sensitivity Wind Speed Sensor Based on Triboelectrification‐Induced Electroluminescence

**DOI:** 10.1002/advs.201901980

**Published:** 2019-09-30

**Authors:** Li Su, Hailu Wang, Zhen Tian, Haojie Wang, Qian Cheng, Wei Yu

**Affiliations:** ^1^ Hebei Key Laboratory of Optic‐electronic Information and Materials National‐Local Joint Engineering Laboratory of New Energy Photoelectric Devices College of Physics Science and Technology Hebei University Baoding City 071002 Hebei Province China; ^2^ CAS Center for Excellence in Nanoscience Beijing Key Laboratory of Micro‐Nano Energy and Sensor Beijing Institute of Nanoenergy and Nanosystems Chinese Academy of Sciences Beijing 100083 China

**Keywords:** high sensitivity, low detection limits, triboelectrification‐induced electroluminescence, wind speed sensors

## Abstract

The rapid development of the Internet of Things (IoT) has tremendously increased the demands for wind speed sensors in various applications, such as weather forecasting and environmental monitoring. To date, many wind speed sensors are developed based on triboelectric nanogenerators (TENGs). However, the low output current leads to a poor sensing precision, which greatly limits their practical applications. Here, a wind speed sensor is proposed by integrating a wind‐driven triboelectrification‐induced electroluminescence (TIEL) component with a perovskite‐based photodetector (PD) for enlarged electric current output. Compared with mechanoluminescence (ML), TIEL displays significantly higher signal intensity even under gentle breezes. In addition, the emission peak of TIEL matches with the absorption band of perovskite materials. Thus, TIEL materials are promising candidates for generating distinct electrical signals in real‐time to facilitate the detection of wind speed. With a comparable detection limit (5 m s^−1^) to a conventional TENG‐based wind speed sensor, the sensitivity of this hybrid sensor is three magnitudes higher at low bias voltages and, the response time is extremely short (<0.3 s). Moreover, it shows a favorable correlation with a commercial sensor. This research provides important progress toward environmentally‐friendly light sources and TIEL‐related sensor systems.

## Graphical abstract

A type of wind speed sensor system is designed and fabricated for wind speed detection by integrating a wind‐driven triboelectrification‐induced electroluminescence (TIEL) component with a perovskite‐based photodetector (PD). This research not only provides essential progress toward an environmentally friendly light source but also demonstrates a practical application of TIEL‐related sensor systems.

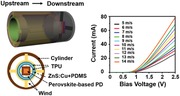

## 1 Introduction

With the rapid development of the Internet of Things (IoT), there is an increasing number of decentralized sensors that are widely applied in daily activities and industrial productions.^1^, ^2^, ^3^, ^4^, ^5^ Among these applications, wind speed sensor plays an essential role in environmental sensing, such as weather forecast and environment monitoring.^6^, ^7^, ^8^ A number of sensors have been developed based on triboelectric nanogenerators (TENGs), which is known for the portability, facile fabrication, and low cost. These TENG‐based sensors have been considered as one of the most effective mechanical energy conversion technologies as they enable the generation of high open‐circuit voltage at a low frequency.^9^, ^10^, ^11^ Particularly, TENG‐based wind speed sensors have been tremendously developed by varying sensing principles, including the wind‐induced rotation,^12^ vertical contact‐separation motions,^13^ and fluttering behavior.^14^ Y. Yang et al.^15^ reported a TENG‐based wind speed sensing system, which displays a sensitivity of 0.09 µA/(m/s) and a detection limit 6 m s^−1^ by utilizing the wind‐induced vibration. Based on an anemometer TENG and a wind vane TENG, J. Y. Wang et al.^16^ presented a wind sensor with a sensing range from 2.7 to 8.0 m s^−1^, which generates an output short‐circuit current of 6.3 µA at wind speed of 6 m s^−1^. In another work, P. H. Wang et al.^17^ demonstrated a triboelectric‐electromagnetic hybrid wind speed sensor with a maximal loading current of 6 µA and a lower detection limit of 3.5 m s^−1^. However, the applications of these traditional TENG‐based wind speed sensors are limited by the extremely low output current at the level of µA. Therefore, strategies for enhancing the output current are in great demand when designing an efficient wind speed sensor.

In this context, we propose a novel wind speed sensor based on a wind‐driven luminescence device and a photodetector (PD), so as to utilize the high output current of the PD. Mechanoluminescence (ML) is the emission of luminescence that is activated by mechanical stress. Due to the low critical wind speed and large amplitude, ML has been induced by wind‐induced flutter vibration on the elastic ZnS:Cu+PDMS (polydimethylsiloxane).^18^, ^19^ However, the application of ML in wind speed sensor is limited by the requirement for high excitation stress, which is at the scale of MPa. S. M. Jeong et al.^20^ reported that the bright white wind‐driven ML could only be observed at speeds higher than 30 m s^−1^, which is at the level of a hurricane or typhoon.

Triboelectrification‐induced electroluminescence (TIEL) has been considered as a novel luminescent which is capable to convert the dynamic motions to luminescent signals by extremely gentle mechanical interactions. It relies on the coupling of triboelectrification and electroluminescence (EL). During the process, tribocharges are generated through the dynamic interactions between two different materials, which further alter the surrounding electric potential (up to hundreds of volts) within milliseconds. This dramatic variation in electric potential excites the EL of the embedded phosphors. TIEL displays significant advantages over ML, such as the low‐stress threshold value, high‐stress responsivity, and the nondestructive property.^21^, ^22^, ^23^ Driven by these advantages, the fabrication of TIEL device has been extensively studied, mainly through metal ion–doped (Cu^+^, Mn^2+^, and others) ZnS particles combing with PDMS elastomeric matrix. By now, TIEL device has been widely applied in real‐time sensing, anticounterfeiting, self‐powered illumination, and human/machine interactive display.^24^, ^25^, ^26^, ^27^

Considering the stress responsivity of TIEL is nearly three orders higher than ML in the low‐stress region,^26^ the wind‐driven TIEL in elastic ZnS:Cu+PDMS should display a significantly improved luminescent property than ML. In addition, wind‐driven TIEL can be integrated with a PD to generate real‐time electrical signals for wind speed detection with an enhanced output current than TENG‐based sensor. Therefore, the proposed hybrid wind speed sensor would not only present a lower detection limit but also present significantly higher output current.

Herein, an effective and robust wind speed sensor based on the wind‐driven TIEL is demonstrated for the first time. First, a multilayered material was constructed with a luminescent layer (ZnS:Cu+PDMS) and two electrification layers (TPU, thermoplastic polyurethane). Under the wind‐induced flutter vibration, the layers repeatedly contacted and separated with each other. Second, the tribocharges caused by the mechanical movement of the layers could generate alternating electric potential, which excited the EL of underlying phosphors in the luminescent layer. In this step, wind energy was converted into TIEL. Next, wind‐driven TIEL was integrated with a perovskite‐based PD to enable the conversion of varying TIEL intensities into real‐time electric signals under adjustable wind speed. This novel wind speed sensor exhibited a low detection limit (5 m s^−1^), an excellent responsivity of 1.72 mA/(m/s) at 1.5 V bias voltage, as well as a rapid response time (<0.3 s). It is also worth noting that, the sensing performance of the sensor present in this work is comparable with a commercial sensor as it is capable to detect wind speed from 5 to 14 m s^−1^. This work presents a key step toward practical application of new environmental‐friendly light source and the TIEL‐related sensor systems.

## 2 Results and Discussion

**Figure 1:**
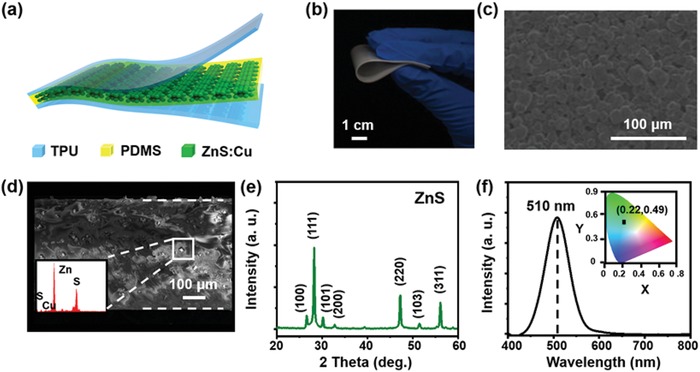
Structure of the TIEL composite material. a) Schematic of the layer‐by‐layer composite material. b) Photograph of a bent luminescence layer (ZnS:Cu+PDMS). c) Surface and d) cross‐sectional SEM images of the luminescent layer displaying the ZnS:Cu particles embedded in a PDMS matrix. Inset: EDS of the phosphor. e) XRD pattern of the phosphors. f) The wind‐driven TIEL spectrum obtained from the composite material. Inset: CIE coordinates (*x*, *y*) corresponding to the TIEL spectrum.

The schematic structure of the TIEL material is shown in **Figure** **1**a. The multilayered material is partially sliced and composed of a luminescent layer prepared with PDMS, ZnS:Cu phosphor particles and two TPU electrification layers. The fabrication procedure is illustrated in the Experimental Section. A piece of the as‐fabricated bent ZnS:Cu+PDMS film is shown in Figure 1b. Figure 1c,d displays the surface and cross‐sectional views of the luminescent layer recorded by a scanning electron microscope (SEM). The result suggests that the ZnS:Cu phosphor particles homogeneously dispersed in the PDMS substrate with an estimated average diameter of 20 µm. The energy dispersive spectroscopy (EDS) was conducted to analyze the elemental compositions of particles within the defined regions as shown in Figure 1d. The results (displayed in the inset) reveal the characteristic peaks of ZnS:Cu. Figure 1e displays the X‐ray diffraction (XRD) pattern of the ZnS:Cu powders. It is clear that three major peaks at 28.82°, 48.31°, and 56.0° are detected, indicating the (111), (220), and (311) lattice planes of the zinc‐blended structure, respectively. The wind‐driven TIEL spectrum of the composite material was detected at the light‐emitting section by a spectrometer. The coordinates corresponding to Commission Internationale de L'Eclairage (CIE) are displayed in Figure 1f. The result shows that a green emission is detected at 510 nm, corresponding to the CIE coordinates (0.22, 0.49). Based on the energy level diagram shown (Figure S1, Supporting Information), electrons in the shallow electron trap states of ZnS: Cu would fall into the states of Cu impurity, which emits a green luminescence through recombination under the mechanical activation.

**Figure 2:**
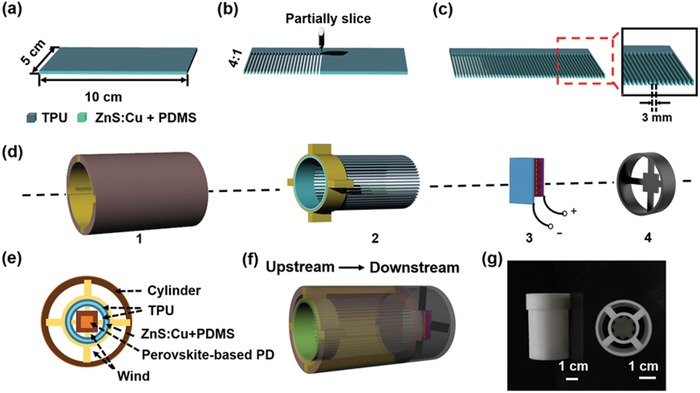
Schematic of the fabrication process of the wind speed sensor. a) The dimension of the TIEL material is 5 × 10 cm^2^. b) The TIEL material is cut into slices with a cut to uncut ratio of 4:1. c) The width of the sliced TIEL material is 3 mm. d) The wind speed sensor consists of four components: 1) a cylinder with four grooves; 2) a hollow ring with four cross‐shaped ridges for adhesion with the TIEL material; 3) a perovskite‐based PD; 4) a round base. e) The front view and f) side view of the wind speed sensor. g) Photograph of the wind speed sensor without the TIEL material.

The fabrication of the wind speed sensor is illustrated in **Figure** **2**. First, the composite materials were sequentially stacked layer by layer on a glass substrate (5 × 10 cm^2^). The ZnS:Cu+PDMS and TPU film respectively serve as the luminescent layer and the electrification layer, respectively (Figure 2a). Second, the film was sliced into two parts, where the ratio of sliced to unsliced is 4:1 along the width direction (Figure 2b). The sliced section was cut into strips with a width of 3 mm to increase the contact area (Figure 2c, the photograph is shown in Figure S2, Supporting Information). The wind speed sensor is composed of four major parts as shown in Figure 2d and was fabricated by the process described in the Experimental Section. A cylinder was used to support the whole device, which not only enclosed the entire structure within a confined channel to conduct the wind but also concentrated the TIEL. A hollow ring with four cross‐shaped ridges was used to adhere to the TIEL materials, which were then inserted into the four grooves of the cylinder. The as‐prepared film was rolled and fixed into the uncut part (upstream side). The rest of the sliced part was isolated from layers and maintained flexible at the downstream, which was followed by a perovskite‐based PD and a round base. Two lead wires were connected to the PD for electrical measurement. Figure 2f,g respectively shows the front and side views of a typical as‐fabricated wind speed sensor. Figure 2h shows the wind speed sensor with a size of 3 × 5 × 3 cm^3^ without TIEL material, which was fabricated by 3D printing techniques.

**Figure 3:**
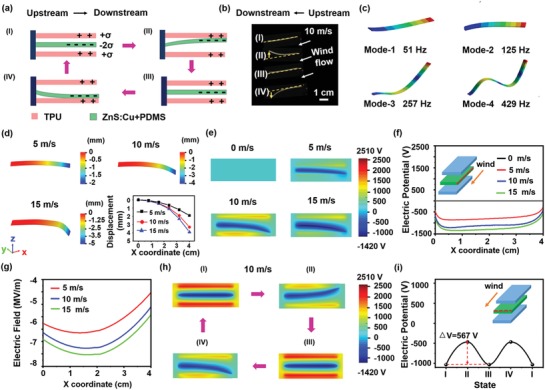
Emission mechanism of the wind‐driven TIEL. a) Schematic diagrams displaying the generation of electricity by the TIEL material resulted from the wind flutter vibration. b) Serial photos showing four vibration states of an oscillating ZnS:Cu film. c) Variant vibration modes of the ZnS:Cu film simulated by ANSYS. d) COMSOL simulation of displacement distribution of the ZnS:Cu film at wind speeds of 5, 10, and 15 m s^−1^. e) Lateral electric potential distribution simulated by COMSOL at wind speeds of 0, 5, 10, and 15 m s^−1^. f) Corresponding electrical potential distribution and g) electric field on the ZnS:Cu film along the dashed line defined in inset (f) at wind speeds of 0, 5, 10, and 15 m s^−1^. h) Lateral electric potential distribution in an intact contact‐separation cycle under four states at a wind speed of 10 m s^−1^. i) Corresponding electric potential distribution along the dashed lines defined in the inset that shows the electric potential difference.

As shown in **Figure** **3**, the working principle of the wind‐driven TIEL involves the coupling of contact electrification and EL. Figure 3a illustrates the process of electricity generation via the TIEL material in a working cycle, including four typical states. For the simplification of demonstration, the ZnS:Cu+PDMS film can be regarded as a flat surface with a vertical motion, while the TPU films are relatively static. At the original position I, the difference between the triboelectric polarizations of PDMS and TPU leads to the generation of opposite charges on their surfaces when the two TPU films and the ZnS:Cu+PDMS film fully contact with each other, followed by the balance of the charges. Under the vibration effect caused by wind flutter, the ZnS:Cu+PDMS film bends up and down, which dynamically changes the electric potential. The potential reaches the maximum value at the largest displacement point (states II and IV) and rapidly drops back to the minimum value in the balance position (states I and III). Thus, a series of voltages can be generated on the surface of the ZnS:Cu+PDMS film which excites TIEL. The oscillatory behaviors of the ZnS:Cu+PDMS film at wind speed of 10 m s^−1^ were captured by a high‐speed camera as shown in Figure 3b (the overall procedure refers to Movie S1, Supporting Information). The frequency of the oscillation is examined to be 16 Hz without adhesion in the oscillation, suggesting that ZnS:Cu+PDMS film displays excellent mechanical behavior. ANSYS software was used to simulate the natural frequencies and modes of the first four order vibration modes. As shown in Figure 3c, the first‐order mode bends at the frequency of 51, which is consistent with the actual vibrations of the ZnS:Cu+PDMS film. To examine the effect of wind flutter vibration, we qualitatively predicted the interaction between wind and the ZnS:Cu+PDMS film under the first‐order mode by COMSOL software. In the simulation, the inlet wind speed was set as 5, 10, and 15 m s^−1^, respectively, with zero pressure at the outlet. The density *F* and the dynamic viscosity η of the airflow were set as 1.225 kg m^−3^ and 1.983 × 10^5^ Pa, respectively. The density *F* of the ZnS:Cu+PDMS film was set at 1420 kg m^−3^ with Young's modulus *E* of 2 M Pa and a Poisson's ratio ν of 0.49.^28^ The model was composed of two parts, including a fluid part (incompressible flow and Newtonian) computed by the Navier–Stokes equations in the flow channel and a solid mechanical part (compressible and elastic). Moreover, an elastic formulation and a linear geometry formulation were used to solve the deformation of the ZnS:Cu film to allow for large deformation. In the solid mechanical part, one edge of the film was set in the middle of the fluid channel wall, which is vertical to the fluid flow streamline. The flowing fluid is borne by all the boundaries of the belt structure. Meanwhile, there was no pressure at the outlet and the rest four surfaces of the fluid channel were set as nonslip, indicating that the surfaces work as walls to restrain the flow. As shown in Figure 3d, the deformation of the ZnS:Cu+PDMS film was simulated under three wind speeds at the inlet, which suggests that the film functions majorly in a bent mode. Consistent with other reports,^29^ the degrees of film deformation are similar to each other as the wind speed increases over 10 m s^−1^, indicating that the ZnS:Cu+PDMS film is stable when wind speed at the inlet reaches a certain value. In addition, under different wind speeds, various deformations result in the changes of electric potentials by wind‐induced vibration effect. COMSOL was then utilized to simulate the distributions of electric potential of the two TPU films and the ZnS:Cu+PDMS film at the wind speed of 0, 5, 10, and 15 m s^−1^, respectively, without external electron flow (an ideal condition). In the process of triboelectrification, TPU is positively charged while PDMS is negatively charged. In order to validate the proposed simulation, the charge density σ on the surface of TPU was set at 100 µC m^−2^, which was measured by an electrostatic voltmeter (model 279, Monroe Electrostatics). The lateral distribution of electric potential is illustrated in Figure 3e. The electric potential and electric field within the luminescent layer are respectively depicted in Figure 3f,g. These results indicate that the electric potential and electric field of ZnS:Cu+PDMS film increase with the displacement, followed by the tendency to maintain stable, which is consistent with the actual deformations as shown in Figure 3d. These results suggest that the displacement determines the effective contact area between the TPU and ZnS:Cu+PDMS surfaces. Meanwhile, the distributions of electric potential between the TPU films and ZnS:Cu+PDMS film are also simulated within a full contact‐separation cycle in the four states at wind speed of 10 m s^−1^ (Figure 3h). Figure 3i shows the recorded potentials with dashed lines indicating the potential difference of repeat changes. It is noteworthy that the potential reaches the maximum value when the displacement reaches the upper limit, while the potential drops to the minimum value at the balance position. Consequently, this ac‐like dynamic variation (Δ*V* = 567 V) excites the luminescence of the phosphors, which further induces the wind‐driven TIEL.

**Figure 4:**
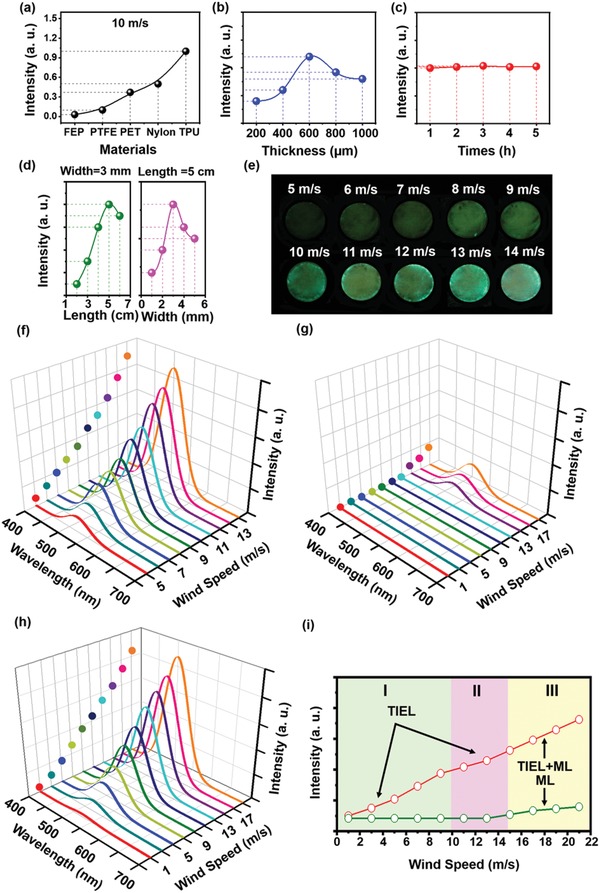
Experimental measurement results of the wind‐driven TIEL. Dependence of wavelength spectra on a) the materials of the electrification layer, b) thickness of the luminescent layer, c) wind blowing time, and d) length–width ratio of the material at a wind speed of 10 m s^−1^. e) Wind‐driven TIEL photographs vertically taken from the bottom of the device under the tunable wind speed from 5 to 14 m s^−1^. f) Corresponding TIEL intensity under the tunable wind speed from 5 to 14m s^−1^. g) ML and h) TIEL intensity at different wind speeds from 1 to 21 m s^−1^. i) Corresponding intensity of ML and TIEL in distinct regions.

The luminescent properties of the wind‐driven TIEL (Movie S2, Supporting Information) are studied in **Figure** **4**. The compressed air is used to generate the wind with an adjustable speed by a pressure regulator in order to simulate natural wind. First, material chosen of the electrification layer is a primary factor, which is fabricated by different polymer films, including fluorinated ethylene propylene (FEP), polytetrafluoroethylene (PTFE), polyethylene glycol terephthalate (PET), Nylon, and TPU. As shown in Figure 4a, the TPU sample has the highest luminescence intensity, followed by the Nylon‐based sample, which can be attributed to different electric polarities of these materials.^30^ The triboelectric polarity of FEP is similar to that of the PDMS‐based material, resulting in the weakest luminescence. The Scanning Kelvin probe microscope (SKPM) has been employed to characterize the surface charge density of the luminescence layer after rubbing with these materials (Figure S3, Supporting Information). The instantaneously increment of the surface charge density is in good agreement with the experimental results (Figure 4a). Thus, in this case, the TPU is selected as the electrification layer because of its positive electricity and excellent mechanical stability. The TIEL mechanism is further proposed by the dependence of above materials on the luminescence intensity, and other mechanism is excluded, such as ML. Second, the impact on the film thickness is discussed in Figure 4b. It is found that if the phosphor layer is excessively thick, the light emission would be blocked, resulting in the decrease of light intensity. The strongest luminescence intensity is obtained with an optimal thickness of 600 µm. Third, durability test was also conducted in Figure 4c. After exposure for 5 h, the intensity of the TIEL basically remains the same, which shows the excellent stability and repeatability of TIEL. Fourthly, the length–width ratio of the material in the slicing process (Figure 2b) is also an important factor to determine the TIEL intensity. On the one hand, if the length–width ratio of the material is too small, the contact areas of strips between the layers are small. On the other hand, a larger length–width ratio would make the system in a complex vibration state and induce the adhesion of the system, thus resulting in a lower TIEL intensity. In this case, the width of the phosphor layer is 3 mm, and the optimal length of the material is 5 cm, which leads to the strongest TIEL intensity (Figure 4d). Furthermore, the photographs of the wind‐driven TIEL under different wind speed (5–14 m s^−1^) are displayed in Figure 4e, which are taken along the direction vertical to cylinder bottom and the luminescence intensity mapping is obtained after image processing by MatLab (Figure S4, Supporting Information). The corresponding TIEL spectra show that the luminescence intensity increases with wind speed increasing, whereas the positions of the emission peaks are almost unchanged (Figure 4f). In addition, materials without the electrification layers (TPU) are prepared under the same condition to study the effect of ML. By comparison, it is found that the lowest excitation wind speed of ML is 15 m s^−1^ (Figure 4g), which is much higher than that of the TIEL (1 m s^−1^) (Figure 4h). This indicates that the ML could be only obtained under a moderate wind, but the TIEL could be successfully obtained under a light wind. Specific values of TIEL and ML under different wind speed are shown in Figure 4i, and a three‐section behavior of the luminescence intensity can be observed (top curve). According to previous reports,^24^ the amplitude and the frequency of the electric potential variation play key roles in the TIEL intensity. In section I, under a low wind speed, the effect of contact electrification plays a decisive role, leading to the enlargement of the electric potential amplitude and the increment of TIEL intensity. In section II, TIEL intensity increasing slows down due to the saturation of the tribocharge density. In this part, the amplitude no longer increases with the increase of wind speed, TIEL intensity is only related to the frequency. In section III, TIEL intensity further increases, which is attributed to the superposition of TIEL and ML. The ML of the phosphors appears as the wind speed exceeds 15 m s^−1^ (bottom curve), which is consistent with previous reports on the threshold stress of ZnS‐based materials.^22^ Nevertheless, although TIEL can be emitted at a speed of 1 m s^−1^, only efficient TIEL excited with a speed more than 5 m s^−1^ can be effectively converted into electrical signals by the perovskite‐based PD. The research orientation is to further improve the TIEL intensity and obtain the stronger luminescence at a lower wind speed.

**Figure 5:**
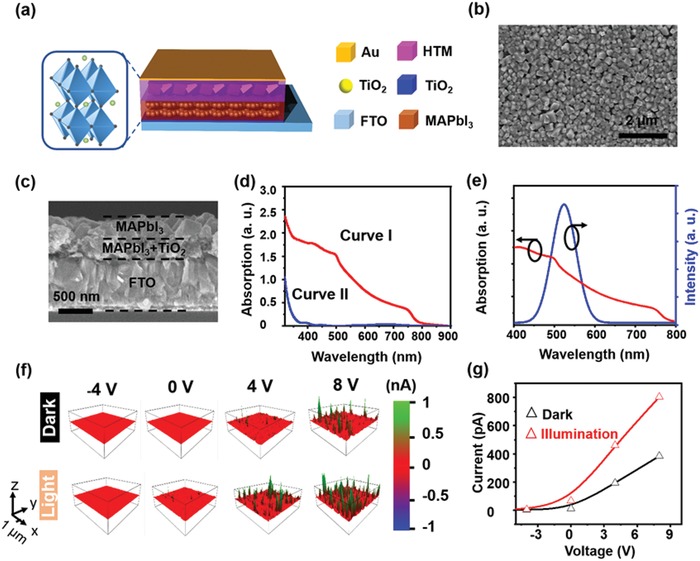
Structure and characterization of the perovskite‐based PD. a) Structure of the device. Inset shows the 3D schematic of the crystal structure of MAPbI_3_. SEM images of b) surface and c) cross‐sectional view of the MAPbI_3_‐based film. d) Absorption spectra of the MAPbI_3_‐based composite film (Curve I) and the mesoporous TiO_2_ film (Curve II). e) Absorption spectrum of MAPbI_3_‐based composite film and its overlapping wavelength with the wind‐driven TIEL emission. f) Current distribution mapping of the PD with bias voltages from −4 to +8 V and increments of 4 V in a dark environment or upon illumination (power density of 50 mW cm^−2^). g) Corresponding *I–V* characteristics of the PD.

Apart from the TIEL intensity, the performance of perovskite‐based PD is also critical to the wind speed sensor. **Figure** **5**a shows that the solution‐processed MAPbI_3_ perovskite thin film that was used in the PD as the light‐absorbing material. The fabrication procedure is illustrated in the Experimental Section. Figure 5b,c respectively displays the surface and cross‐sectional images taken by SEM. It is shown that the TiO_2_ layer (300 nm in thickness) on the TiO_2_ hole blocking layer (25 nm in thickness) is filled with crystalline MAPbI_3_ and covered by a layer of MAPbI_3_ nanoparticles with a diameter of 200 nm. The average surface of roughness is 28 nm, which is determined by the atomic force microscopy (AFM) measurement (Figure S5a, Supporting Information). The XRD pattern is according with those reported in the previous tetragonal perovskite structure (Figure S5b, Supporting Information).^31^ From the UV–vis absorption spectrum in Figure 5d, the absorption edge of the MAPbI_3_‐TiO_2_ composite film (Curve I) is at 760 nm, which also agrees with previous reports.^32^ Compared with the compact TiO_2_ (Curve II), there is a broad range for the absorption of the MAPbI_3_‐TiO_2_ composite film. Furthermore, as shown in Figure 5e, the absorption band of the perovskite material largely overlaps with the emission band of ZnS:Cu, indicating that the PD is able to absorb the TIEL and convert it into electric signals and enable the detection of the wind speed. Lastly, conductive atomic force microscopy (CAFM) in a contact mode is employed to illustrate the photoresponse property of the perovskite‐based PD. Figure 5f shows the current distribution mapping under the illumination of 50 mW cm^−2^. Clearly, the current through the illuminated sample is considerably higher than that in dark environment. The quantitative analysis in Figure 5g indicates that the excited sample with a bias voltage of 8 V shows a photocurrent of 813 pA, which is 2.5‐fold higher than it is in a dark environment (325 pA). Therefore, these results suggest that the perovskite‐based PD displays an excellent photoresponse property for rapid detections of various light signals.

**Figure 6:**
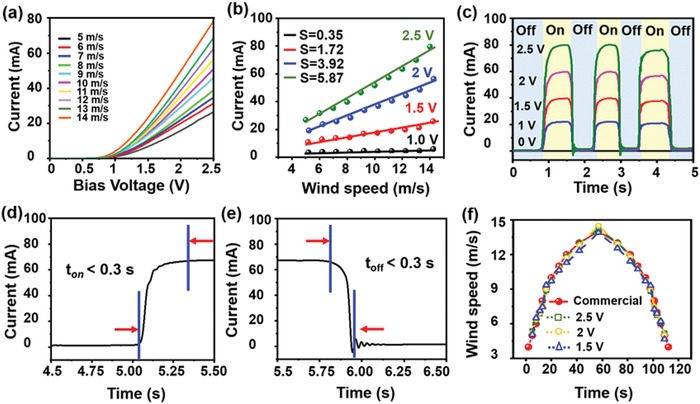
Performance of the wind speed sensor. a) *I–V* characteristics of the wind speed sensor under tunable wind speed. b) Current dependence of the wind speed changes and the corresponding linear fitting curves. c) Changes of current upon repeated wind‐driven TIEL (14 m s^−1^) with different bias voltages. d) Photocurrent increase and e) decrease of the wind speed sensor measured with a bias of 2 V at a wind speed of 14 m s^−1^. f) Real‐time wind speed curves detected by a commercial wind speed sensor and a fabricated wind sensor system.

In the last section of this paper, the sensing performance of the wind speed sensor is investigated by measuring the efficiency of converting wind energy into the electric signals. **Figure** **6**a shows the *I–V* output characteristics of the PD from 0 to 2.5 V under a range of wind speed from 5 to 14 m s^−1^. As shown in Figure 6b, the change of the current amplitude under four bias voltages (1.0, 1.5, 2.0, and 2.5 V) are plotted as a function of wind speeds and the average sensitivity values are determined to be 0.35, 1.72, 3.92, and 5.87 mA/(m/s), respectively, by the linear fitting. In terms of the signal intensity, it is three orders higher than the signals of recently reported TENGs‐based wind speed sensors. In addition, the response time, representing a significant parameter of a wind speed sensor, is quantitatively analyzed as shown Figure 6c, which records the photocurrent of the wind speed sensor during repetitive switching of wind flow (14 m s^−1^) within 0–5 s. Here, the rise time and decay time are defined as the time spent for the initial current increases to 90% of the peak value, and vice versa. As shown in Figure 6d,e, it is clear that both the rise time and decay time are both less than 0.3 s. Therefore, this highly stable and rapidly responsive device is promising to be utilized as a wind speed sensor, which is superior to TENGs. In addition, the sensing performance of the system was further characterized in a mimetic environment that simulates the flow of natural wind. The real‐time current was obtained from the electrical output of the perovskite‐based PD part as shown in Figure 6f. The wind speed was analyzed based on the correlation between the current and the wind speed at the inlet. In addition, a commercial hot‐wire anemometer was also tested for comparison. It is shown that the wind speed obtained by the current value agrees well with the result of hot‐wire anemometer, suggesting that the wind sensor system enable the instantaneous and stable detection of wind speed, which is expected to be applicable in the development of the IoT and climate monitoring.

## 3 Conclusions

In summary, an effective and robust wind speed sensor is fabricated by integrating a wind‐driven TIEL component and a perovskite‐based PD for the first time. It shows a low detection limit (5 m s^−1^), high output current (≈mA), and rapid response (<0.3 s). Moreover, the sensing performance of this device is significantly superior to the existing TENG‐based wind speed sensor and is also comparable with a commercial sensor. Therefore, this study presents a key step for the practical application of new environmental‐friendly light source and the sensor systems related to TIEL.

## 4 Experimental Section

*Fabrication of TIEL Material*: The EL ZnS: Cu was purchased from Ke Yan Company. TPU film was purchased and had a thickness of 100 µm. Acrylic substrates with a size of 10 × 5 × 0.6 cm^3^ were prepared by laser cutting. The ZnS:Cu particles were mixed with PDMS at a ratio of 1:5 to form a homogenized paste, which was then coated on the acrylic substrate. The coated substrate was dried at 80 °C for 3 h, after which the PDMS layer was peeled off from the substrate. Two identical layers of TPU were pasted on the front and back of the as‐prepared ZnS:Cu+PDMS film before being sliced.

*Fabrication of the Wind Speed Sensor*: The wind speed sensor was composed of four parts, including a cylinder, a hollow ring with four cross‐shaped ridges for adhesion, a perovskite‐based PD, and a round base. Except for the TIEL material, the other parts were all fabricated by 3D printing technology with resin material. The sizes of the cylinder, four cross‐shaped ridges, and the hollow ring were 5 × 3 × 0.2 cm^3^, 1 × 0.75 × 0.4 cm^3^, and 1 × 1.5 × 0.2 cm^3^, respectively. The inlet and outlet for the wind to flow through the device were 6.3 and 4.4 cm^2^, respectively. The area of perovskite‐based PD was 1.2 × 1.2 cm^2^, which was fixed onto the round base with four channels for wind convection.

*Fabrication of Perovskite‐Based PD*: The perovskite‐based PD was composed of SnO_2_:F (FTO) conductive glass, compact TiO_2_, mesoporous TiO_2_, MAPbI_3_ light absorption layer, MAPbI_3_ crystalline capping layer, hole transport layer (HTL), and gold electrode in sequence. First, FTO conductive glass was cleaned by detergent, followed by ultrasonic wave with acetone and ethanol. Then, a compact TiO_2_ layer (25 nm in thickness) was coated onto the cleaned FTO substrate by 500 cycles of atomic layer deposition at a temperature of 200 °C (Picosun Sunaler‐100). TiCl_4_ and N_2_ were respectively utilized as the Ti source and purging gas. TiO_2_ paste (Dyesol‐18NRT) was diluted by ethanol with a weight ratio of 2:7 and the mixture was spin‐coated onto the compact TiO_2_ layer at 5500 rpm. The coated TiO_2_ layer was sintered at 500 °C for 30 min during which a mesoporous TiO_2_ layer was formed with a thickness of 300 nm. Pbl_2_ in *N*,*N*‐dimethylformamide solution (462 mg mL^−1^) was infiltrated into the mesoporous TiO_2_ layer by spin coating at 6500 rpm, followed by drying at 70 °C. The dried TiO_2_ layer was then dipped into an MAPbI_3_ solution (9 mg mL^−1^ in 2‐propanol) for 30 s and sintered at 70 °C for 30 min. The concentration of the CH_3_NH_3_I in the solution was adjusted to control the crystal size of the MAPbI_3_ capping layer. The HTL was deposited by spin‐coating at 3000 rpm for 30 s with Spiro‐OMeTAD solution. This solution was obtained by dissolving 73.5 mg Spiro‐OMeTAD powders in 1 mL chlorobenzene with 28.8 µL tBP and 17.5 µL Li‐TFSI in acetonitrile (520 mg L^−1^). Lastly, the gold electrode layer was deposited by e‐beam evaporation.

*Measurements*: The crystal structure was characterized by XRD (x pert 3). All SEM and EDS images were recorded with the SU8020 camera. The absorption properties were analyzed by the UV–vis–NIR light source (Shimazu UV‐3600). Optical emission was recorded by a spectrometer and optical fibers arranged perpendicularly with collimating lenses (NOVA). All the electrical measurements were performed with the Keithley 2400 system electrometer. The surface morphology investigation, the SKPM, and CAFM measurements were carried out with the MFP‐3D AFM in an ambient environment (Park Systems NX‐10). The Ir‐based probe with Pt coating was obtained from Nanoworld (product no. EFM‐10).

## Conflict of Interest

The authors declare no conflict of interest.

## Supporting information

Supplementary

Supplementary

Supplementary
