# Supplementary material for: Low Detection Limit and High Sensitivity Wind Speed Sensor Based on Triboelectrification‐Induced Electroluminescence
Source: Adv Sci (Weinh). 2019 Sep 30;6(23):1901980. doi: 10.1002/advs.201901980 (PMC6891903; doi:10.1002/advs.201901980)
Supplement: Supplementary file 1 — Supplementary [file ADVS-6-1901980-s002.pdf]

Copyright WILEY-VCH Verlag GmbH & Co. KGaA, 69469 Weinheim, Germany, 2019.

Supporting Information

for *Adv. Sci.*, DOI: 10.1002/advs.201901980

Low Detection Limit and High Sensitivity Wind Speed Sensor Based on Triboelectrification-Induced Electroluminescence

Li Su, Hailu Wang, Zhen Tian, Haojie Wang, Qian Cheng, and Wei Yu**

Copyright WILEY-VCH Verlag GmbH & Co. KGaA, 69469 Weinheim, Germany, 2019.

Supporting Information

Low Detection Limit and High Sensitivity Wind Speed Sensor Based on Triboelectrification-induced Electroluminescence

Li Su, Hailu Wang, Zhen Tian, Haojie Wang, Qian Cheng, and Wei Yu**

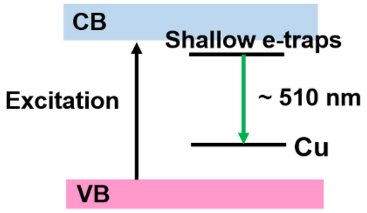

Figure S1. Band diagram of the EL of ZnS:Cu phosphor.

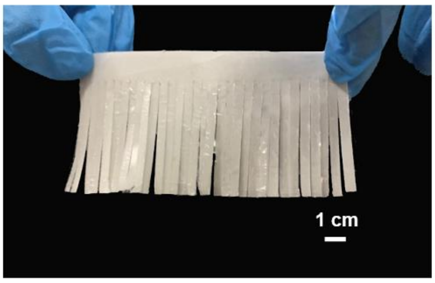

Figure S2. Photograph of the sliced TIEL composite material.

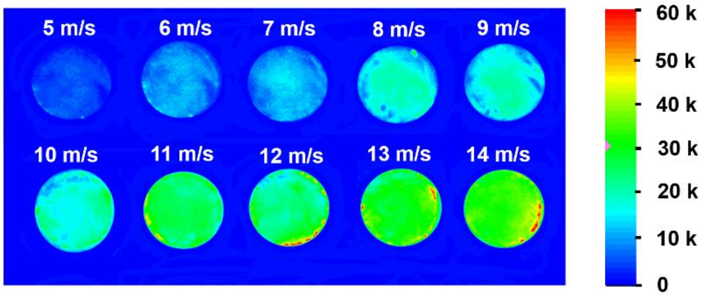

Figure S3. The wind-driven TIEL intensity mapping recorded along the vertical direction to the cylinder bottom.

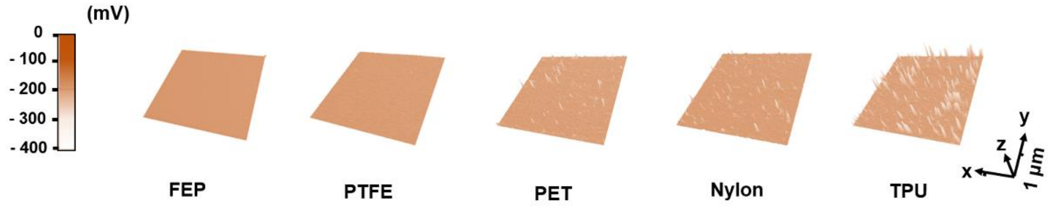

Figure S4. SKPM 3D mappings of the surface potential of the luminescence layer (ZnS:Cu+PDMS) after the rubbing with the polymers.

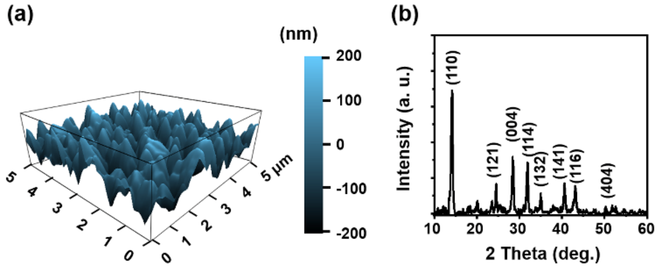

Figure S5. a) Morphology of the $MAPbI_3$ capping layer recorded by AFM. b) XRD diffraction pattern of the $MAPbI_3$-based film on FTO glass.

Movie S1. The dynamic process of the oscillation behavior of ZnS:Cu+PDMS film at a wind speed of 10 m/s.

Movie S2. The wind-driven TIEL at a wind speed of 10 m/s.
